# Antibodies of the immunoglobulin a isotype to novel antigens in early axial spondyloarthritis

**DOI:** 10.3389/fmed.2022.1072453

**Published:** 2023-02-08

**Authors:** Pieter Ruytinx, Patrick Vandormael, Dana Quaden, Elien Luyten, Piet Geusens, Johan Vanhoof, Anouk Agten, Frank Vandenabeele, Kurt de Vlam, Veerle Somers

**Affiliations:** ^1^UHasselt, Department of Immunology and Infection, Biomedical Research Institute, Diepenbeek, Belgium; ^2^ReumaClinic, Genk, Belgium; ^3^Maastricht University Medical Center, Maastricht, Netherlands; ^4^UHasselt, Faculty of Rehabilitation Sciences, REVAL-Rehabilitation Research Center, Diepenbeek, Belgium; ^5^Department of Rheumatology, University Hospitals Leuven, Leuven, Belgium; ^6^Department of Development and Regeneration, Skeletal Biology and Engineering Research Center (SBE), Katholieke Universiteit Leuven, Leuven, Belgium

**Keywords:** biomarkers, axial spondyloarthritis (axSpA), diagnosis, isotype, antibodies

## Abstract

**Introduction:**

There is an unmet need for biomarkers to identify patients with axial spondyloarthritis (axSpA). Increasing evidence suggest the presence of autoantibodies in a subset of axSpA patients. The aim of this study was to identify novel IgA antibodies in early axSpA patients and to determine their diagnostic potential in combination with previously determined IgG antibodies against UH (Hasselt University)-axSpA-IgG antigens.

**Methods:**

An axSpA cDNA phage display library constructed from axSpA hip synovium, was used to screen for novel IgA antibodies in plasma from early axSpA patients. The presence of these antibodies against novel UH-axSpA-IgA antigens was determined in two independent axSpA cohorts, in healthy controls and in patients with chronic low back pain.

**Results:**

We identified antibodies to 7 novel UH-axSpA-IgA antigens, of which 6 correspond to non-physiological peptides and 1 to the human histone deacetylase 3 (HDAC3) protein. IgA antibodies against 2 of these 7 novel UH-axSpA-IgA antigens and IgG antibodies against 2 of the previously identified antigens were significantly more present in early axSpA patients from the UH cohort (18/70, 25.7%) and the (Bio)SPAR cohort (26/164, 15.9%), compared to controls with chronic low back pain (2/66, 3%). Antibodies to this panel of 4 antigens were present in 21.1% (30/142) of patients with early axSpA from the UH and (Bio)SPAR cohorts. The positive likelihood ratio for confirming early axSpA using antibodies to these 4 UH-axSpA antigens was 7.0. So far, no clinical correlation between the novel identified IgA antibodies and inflammatory bowel disease could be identified.

**Discussion:**

In conclusion, screening an axSpA cDNA phage display library for IgA reactivity resulted in the identification of 7 novel UH-axSpA-IgA antigens, of which 2 show promising biomarker potential for the diagnosis of a subset of axSpA patients, in combination with previously identified UH-axSpA-IgG antigens.

## Introduction

Axial spondyloarthritis (axSpA) is a chronic rheumatic inflammatory disease, which mainly affects the sacroiliac joints and spine. Despite predominant axial involvement, also peripheral joints, entheses and extra-articular tissues including the eyes, gut and skin can be affected (1). AxSpA patients can be either classified as radiographic SpA, traditionally known as ankylosing spondylitis (AS), or as non-radiographic SpA. Whereas AS is a form of axSpA with structural damage to the sacroiliac joints visible on radiographs, non-radiographic axSpA is used to classify patients where such possible damage is not visible using X-ray imaging (1).

Currently, clinical diagnosis of axSpA by the rheumatologist is based on a combination of signs and symptoms. Persistent inflammatory low back pain is the main hallmark of the disease, often accompanied by peripheral joint manifestations including arthritis, enthesitis and dactylitis and/or extra-articular manifestations such as psoriasis, uveitis, urogenital inflammation and inflammatory bowel disease. In the absence of diagnostic criteria, the Assessment of Spondyloarthritis International Society (ASAS) classification criteria are often applied for diagnostic purposes in patients suffering from chronic low back pain (CLBP) and with suspicion of SpA (2). These criteria combine physical examination, presence of sacroiliitis on imaging and laboratory tests for human leukocyte antigen B27 (HLA–B27) and C-reactive protein (CRP) (2, 3). At the early disease phase, it remains very challenging to distinguish axSpA patients from persons with non-inflammatory CLBP. Even despite the availability of magnetic resonance imaging (MRI), a diagnostic delay for patients with suspected axSpA of 5 to 7 years is observed (4, 5). As a consequence, early treatment initiation is often impeded for many patients, which highlights the urgent need for novel objective biomarkers.

In the last decade, the involvement of the humoral component of the immune system in axSpA patients has received increasing support, as antibodies against several microbial, inflammatory, structural and rheumatic antigen targets have been described (6). Data from our research group showed that immunoglobulin G (IgG) antibodies to 3 novel Hasselt University (UH)-axSpA-IgG antigens were significantly more present in early axSpA patients (14%) compared to patients with CLBP (5%) (7). Besides IgG isotype antibodies, an emerging body of evidence indicates the presence of IgA antibodies in axSpA patients. First, increased levels of total IgA were reported in patients with AS compared to healthy controls (8, 9). Those antibodies might be secreted by inflamed mucosal surfaces, as a strong association between gut inflammation and axSpA has been found (10). Indeed, inflammatory bowel disease (IBD) is observed in 6 to 14% of AS patients and subclinical gut inflammation has even been reported in 50% of SpA patients (11). Moreover, an altered humoral immune response has been observed against several microbial pathogens, with increased serum levels of IgA and IgG antibodies against *Klebsiella pneumonia* and *Saccharomyces cerevisae* found in AS patients compared to healthy controls (12–14). The observation that antigenic stimulation in the gut is a possible causative event in AS is further underscored by the finding that sulfasalazine can normalize IgA levels and reduce the activation of the intestinal immune system (15). Lastly, one of the best described autoantibody biomarkers for axSpA are IgA isotype antibodies against the MHC class II histocompatibility antigen invariant (gamma) chain, also known as CD74 (16), which has been confirmed in different studies. De Winter et al. reported on a sensitivity of 55% in early axSpA patients and a corresponding specificity of 63% in persons with chronic back pain (17), which was in line with the recently reported sensitivity of 60% in the BelGian Inflammatory Arthritis and spoNdylitis cohorT (Be-Giant) (18), whereas Riechers et al. reported a sensitivity of 47% in patients with non-radiographic axSpA (19). Additionally, IgA antibodies against CD74 were found to be associated with structural damage in the sacroiliac joints and the spine of axSpA patients in the ENRADAS study cohort (20). On the other hand, no association between such antibodies and microscopic gut inflammation was found in the Be-Giant cohort (18).

The aim of this study was to identify novel IgA antibodies using the serological antigen selection (SAS) procedure. In this approach, a complementary DNA (cDNA) phage display library, expressing axSpA synovial antigens and non-physiological peptides, was screened for IgA antibody reactivity in plasma of early axSpA patients. Antibody reactivity against novel UH-axSpA-IgA antigens was determined in patients with early axSpA from 2 independent cohorts and controls. Additionally, we investigated whether the presence of antibodies against a combination of these novel UH-axSpA-IgA antigens and previously determined UH-axSpA-IgG antigens could further enhance the biomarker potential for early axSpA diagnosis.

## Materials and methods

### Patients and controls

Plasma or serum was collected from patients with axSpA in the ReumaClinic (Genk, Belgium) (UH cohort; *n* = 85), and in the University Hospitals Leuven (Belgium) [(Bio)SPAR, Spondyloarthritis (Biologics) cohort; *n* = 164] (21, 22). All axSpA patients from the UH cohort (*n* = 85) and 72 patients from the (Bio)SPAR cohort had a maximum time of 5 years from diagnosis to study entry, and these patients were considered to have early axSpA. Plasma from patients with non-specific chronic low back pain (CLBP) (*n* = 66) and healthy controls (HC) (*n* = 185) was collected at UH. Plasma from RA patients (*n* = 60) was collected at the ReumaClinic.

AxSpA patients were diagnosed by their treating rheumatologist and classified according to the ASAS classification criteria (2). CLBP was diagnosed according to the European guidelines for the management of non-specific chronic low back pain (23). RA patients fulfilled the 1987 American College of Rheumatology Criteria (24).

This study was conducted in accordance with the Declaration of Helsinki and was approved by the local Ethics Committees of Jessa Hospital, UH, and Ziekenhuis Oost-Limburg (approval no. B243201422699). Biologic samples from patients and healthy controls were previously collected in different studies, which were approved by the Ethics Committees of Jessa Hospital, UH, and University Hospitals Leuven (approval nos. B322201215165, B243201627373, B32220083429, and B32220084074). All patients and healthy controls provided written informed consent, and all human biologic materials used in this study were kindly provided by the University Biobank Limburg (UBiLim) (25) and the Biobank of University Hospitals Leuven.

### Identification of novel antibodies by serologic antigen selection

Serological antigen selection (SAS) is a screening procedure that uses cDNA phage display to identify antigenic targets of novel antibodies of interest. In this study, an axSpA cDNA phage display library previously constructed from synovial hip tissue from 3 different axSpA patients (7), was used to identify novel UH-axSpA-IgA antigens bound by IgA isotype antibodies in pooled plasma from 10 early axSpA patients (early axSpA SAS pool), using the previously described SAS procedure (7). The peptide or protein antigens displayed on the surface of phage clones isolated using the SAS approach, were identified by sequencing the fusion of M13 gene VI with the cDNA inserts (26). The custom-made DNAnalyzer software (7), an Anaconda Python–based multiprocessing program using Biopython (27), allowed automation of the comparison of nucleotide and amino acid sequences to human sequences with the BLAST tool of NCBI (28).

### Clinical characteristics of groups of interest

A flow chart summarizing the different steps of our screening and validation, and the corresponding patient and control populations is shown in Supplementary Figure 1. The SAS method uses the antigens from an axSpA cDNA phage display library to screen for novel antibodies of the IgA isotype in pooled plasma of early axSpA patients. This early axSpA SAS pool consisted of plasma samples from 10 axSpA patients from the UH cohort, which were the same as in our previous screening for novel IgG axSpA antibodies (7). These patients did not receive biological therapy, had a mean ± SD disease duration of 1.4 ± 0.5 years, a mean ± SD age of 40.6 ± 11.8 years, 5 were male (50%), and 8 (80%) were HLA–B27 positive. In the SAS procedure, counterselection was performed using pooled plasma from HC, consisting of 10 healthy control subjects who were age- and gender-matched to the axSpA patients (mean ± SD age of 40.6 ± 12.3 years, 5 (50%) were male).

In order to select the best antigenic targets, antibody reactivity toward the identified, individual UH-axSpA-IgA antigens was determined in additional plasma pools from early axSpA patients and HC. To this end, plasma from 60 additional early axSpA patients from the UH cohort was pooled into 6 plasma pools, each consisting of 10 axSpA patients, and plasma from 30 additional HC was pooled into 3 HC plasma pools of 10 HC each. These 60 selected patients with early axSpA had a mean ± SD age of 42.8 ± 11.6 years, a mean ± SD disease duration of 3.1 ± 1.4 years, 37 (62%) were male, and 31 (51.7%) were HLA–B27 positive. The 30 HC had a mean ± SD age of 46.6 ± 18.9 years, and 15 (50%) were male.

Antibody reactivity against 7 UH-axSpA-IgA antigens identified using SAS was then determined in individual plasma samples from 70 early axSpA patients from the UH cohort (55 axSpA patients from the plasma pools and 15 additional axSpA patients), and 88 age- and gender-matched HC (9 HC from the plasma pools and 79 additional HC, referred to as HC cohort 1). Clinical characteristics of these 70 early axSpA patients are shown Table 1. The 88 HC, referred to HC cohort 1 had a mean age of 44.4 ± 11.9 years and 48 (54.4%) were male.

**TABLE 1:** Characteristics of axSpA patients from the UH cohort and the (Bio)SPAR cohort.

Clinical characteristics	UH cohort (*n* = 70)^†^	(Bio)SPAR cohort	
		Early axSpA (*n* = 72)^‡^	Total axSpA (*n* = 164)^§^
Age (years), mean (SD)	43.0 (12.1)	36.2 (11.8)	41.9 (12.8)
Gender (male), n (%)	39 (55.7)	44 (61.1)	108 (65.9)
HLA-B27 positive, n (%)	38 (56.7)	52 (72.2)	118 (72.0)
Disease duration^a^ (years), mean (SD)	2.8 (1.3)	1.4 (1.7)	10.6 (10.9)
No medication use^b^, n (%)	11 (15.7)	2 (2.8)	10 (6.1)
NSAID use, n (%)	50 (71.4)	44 (80.0)	74 (71.8)
cDMARD use, n (%)	27 (38.6)	13 (23.6)	22 (21.4)
bDMARD use, n (%)	16 (22.9)	30 (41.7)	90 (54.9)
BASDAI, mean (SD)	4.6 (2.0)	4.1 (2.3)	4.5 (2.2)
Active disease (BASDAI > 4), n (%)	28 (53.8)	25 (34.7)	55 (33.5)
BASFI, mean (SD)	4.1 (2.5)	3.0 (2.4)	3.7 (2.8)
ESR (mm/h), mean (SD)	11.8 (15.8)	19.7 (22.2)	19.3 (21.9)
CRP (mg/L), mean (SD)	5.8 (8.4)	13.0 (19.4)	10.9 (16.2)
Extra-articular manifestations^c^, n (%)	14 (20.0)	17 (23.6)	34 (20.7)

^a^Disease duration, time between diagnosis, and blood sampling.

^b^At time of blood sampling.

^c^Including uveitis, inflammatory bowel disease, and psoriasis.

^†^Values were available for all characteristics except for HLA-B27 (*n* = 67), BASDAI (*n* = 52), BASFI (*n* = 51), ESR (*n* = 60), and CRP (*n* = 62).

^‡^Values were available for all characteristics except for HLA-B27 (*n* = 61), NSAID use (*n* = 55), cDMARD use (*n* = 55), BASDAI (*n* = 53), BASFI (*n* = 54), ESR (*n* = 69), and CRP (*n* = 70).

^§^Values were available for all characteristics except for HLA-B27 (*n* = 136), NSAID use (*n* = 103), cDMARD use (*n* = 103), BASDAI (*n* = 99), BASFI (*n* = 92), ESR (*n* = 160), and CRP (*n* = 142).

axSpA, axial spondyloarthritis; BASDAI, Bath Ankylosing Spondylitis Disease Activity Index; BASFI, Bath Ankylosing Spondylitis Functional Index; bDMARD, biological disease modifying anti-rheumatic drug; cDMARD, conventional disease modifying anti-rheumatic drug; CRP, C-reactive protein; ESR, erythrocyte sedimentation rate; HLA-B27, human leukocyte antigen B-27; NSAID, non-steroidal anti-inflammatory drug; SD, standard deviation; UH, University Hasselt.

Thereafter, antibody reactivity against 3 selected UH-axSpA-IgA antigens (UH-axSpA-IgA.1, UH-axSpA-IgA.3, and UH-axSpA-IgA.10) and 2 previously identified UH-axSpA-IgG antigens (UH-axSpA-IgG.4 and UH-axSpA-IgG.8) (7) was determined in individual plasma samples from 70 early axSpA patients from the UH cohort, in 164 axSpA patients from the (Bio)SPAR cohort, 66 persons with CLBP, and an additional expanded set of 109 HC from the UH cohort, referred to as HC cohort 2 (17 HC from HC cohort 1 and 92 additional HC). Clinical characteristics of the axSpA patients from the (Bio)SPAR cohort are shown in Table 1. Healthy controls of HC cohort 2 (*n* = 109) had a mean ± SD age of 43.1 ± 17.7 years and 56 (48.7%) were male. Patients with CLBP (*n* = 66) had a mean ± SD duration of low back pain complaints of 10.3 ± 9.2 years, had a mean ± SD age of 45.3 ± 10.5 years, and 28 (42.4%) were male. Two (3.0%) were smokers, 55 (83.3%) were workers, 30 (45.5%) underwent previous rehabilitation and 12 (18.2%) used medication. The average results (mean ± SD) of questionnaires for pain perception [Numeric Pain Rating Scale (NPRS)], low back pain disability [Modified Oswestry Index (MODI)], physical disability [Physical Activity Scale for Individuals with Physical Disability (PASIPD)] and spinal pain disability [Million’s Visual Analogue Scale (MVAS)] were respectively 5.5 ± 1.7, 9.5 ± 5.4, 12.7 ± 9.2, and 9.2 ± 0.9. For CLBP patients, missing values were below 10%. For the axSpA patients, missing values were below 10% except for BASDAI (27.8%), BASFI (29.1%), ESR (13.9%), CRP (11.4%).

### Phage ELISA

Antibody reactivity against UH-axSpA-IgA antigens displayed on phage particles was measured by phage ELISA in pooled or individual plasma samples as described previously (7, 29) with some minor modifications. In brief, half area 96-well Microlon high-binding microplates (Greiner Bio-One) were coated overnight at 4°C with 4.0 μg/ml anti-M13 mouse monoclonal antibody (clone MM05T, Sino Biological) diluted in coating buffer (0.2M sodium carbonate bicarbonate buffer, pH 9.6). Coated plates were blocked with 5% skimmed milk powder in phosphate buffered saline pH 7.4 (MPBS) for 2 h at 37°C while shaking. Then, plates were incubated with 7 × 10^11^ colony forming units/ml (diluted in 5% MPBS) of phage particles expressing the antigen of interest (specific phage), or phage particles without antigen (empty phage), for 1 hr at 37°C and 30 min at RT. Bound phage particles were then incubated with pooled or individual plasma samples diluted 1/100 in 5% MPBS for 1 hr at 37°C and 30 min at RT, followed by incubation for 1 hr at RT with cross-adsorbed, horseradish peroxidase–conjugated goat anti-human IgA-Fc (Bethyl Laboratories), diluted 1:2,500 in 5% MPBS.

Antibody reactivity against UH-axSpA-IgG antigens was measured as described previously (7).

Within each phage ELISA experiment, samples were tested in duplicate, and experiments were performed independently at least two times. For the pooled plasma samples, antibody reactivity against each phage-displayed UH-axSpA antigen is expressed as the difference (delta) of the average optical density (OD) signal using the respective phage-displayed antigen, and the average OD signal using the phage without antigen. For individual plasma samples, antibody reactivity against a phage-displayed UH-axSpA antigen is expressed as the average ratio of the optical density (OD) signal using the respective phage-displayed antigen, over the OD signal using the phage without antigen [OD (specific phage)/OD (empty phage)]. The coefficient of variation for duplicate ODs, and for ratios of experimental repeats was lower than 20%. Serum or plasma samples resulting in an OD signal higher than 0.5 using the empty phage, were excluded from the analysis.

For each UH-axSpA antigen, a cutoff value for seropositivity was calculated as the mean of the antibody reactivity in the matching HC population plus 3 times SD (after single exclusion of outliers using the same formula). Antibody reactivity against a panel of antigens included antibody-positivity for at least one of the antigens included in the panel.

### Statistical analysis

All statistical analyses were performed using SAS JMP Pro version 14.2, and *p*-values less than 0.05 were considered significant.

The presence of antibodies to particular UH-axSpA antigens, or panels of antigens, was compared between axSpA patients and controls by applying Fisher’s exact test. Continuous clinical characteristics between antibody-positive and antibody-negative axSpA patients were compared using Student’s *t*-tests for variables with a parametric distribution, and Wilcoxon Rank sum test for variables with a non-parametric distribution, whereas categorical characteristics were analyzed by Fisher’s exact tests.

The positive likelihood ratio (LR+ = sensitivity/100-specificity) for confirming a diagnosis of axSpA based on the presence of antibodies against particular UH-axSpA antigens, or panels of antigens, was calculated based on their presence in axSpA patients and persons with chronic low back pain, unless indicated otherwise.

## Results

### Identification of novel IgA antibodies in early axSpA patients

A human axSpA cDNA phage display library containing 1.88 × 10^6^ recombinant clones was previously constructed from synovial hip tissue from 3 axSpA patients (7). Using this large collection of antigens to screen for IgA antibody reactivity in pooled plasma from 10 early axSpA patients, resulted in identification of IgA antibodies against 173 novel antigens. A flow chart summarizing the different steps of our screening and validation, and the corresponding patient and control populations is shown in Supplementary Figure 1.

Antibody reactivity against each of these 173 phage-displayed antigens was first determined in 6 additional plasma pools, each consisting of 10 early axSpA patients, and in 3 additional plasma pools, consisting of 10 HC each. Of the 173 antigens, 84 did not show reactivity in the axSpA plasma pools and were excluded from further analysis. The other 89 phage-displayed antigens were ranked based on the highest reactivity in the number of axSpA plasma pools combined with minimal reactivity in the healthy control pools (Supplementary Table 1). Based on this ranking, seven candidate antigens were selected for measuring antibody reactivity on individual plasma samples, and were annotated UH-axSpA-IgA.1 through UH-axSpA-IgA.10 (UH-axSpA-IgA isotype.target number).

### Identity of UH–axSpA antigens targeted by novel IgA axSpA antibodies

Nucleotide and amino acid sequences of the 7 selected UH–axSpA antigens recognized by IgA antibodies were compared to human and microbial sequences using the custom DNAnalyzer program (Table 2). Analysis of UH-axSpA-IgA.10 showed that its cDNA, consisting of the 3′-coding region of the human histone deacetylase 3 (HDAC3) gene, was fused in the correct reading frame to M13 phage gene VI, which resulted in the expression of the last 22 C-terminal amino acids from the 428 amino acids of the human HDAC3 protein. The 6 other antigens resulted from out-of-frame fusion, or fusion to non-coding sequences (Table 2). As a result, the expressed antigens corresponded to novel non-physiological peptides, between 9 and 53 amino acids in length. These antigens probably comprise mimotopes (30, 31), and showed partial homology to several human proteins.

**TABLE 2:** Identity of 7 novel antigens targeted by IgA antibody responses in early axSpA patients.

Antibody targets	cDNA identity (NCBI accession no.)	Fusion type^a^, in frame^b^	Antigen sequence corresponding to cDNA insert^c^	Size (aa)^d^	Homology on amino acid level (UniProt accession no.)
UH-axSpA-IgA.1	Phosphoribosyl transferase domain containing 1 (NM_001282786.1)	mRNA, coding, no	(A)GETWPGAARR RQTTGEAS*	19	***Human proteins*** 8/10 (80%) Kinesin light chain 3, *KLC3* (Q6P597) 9/13 (69%) Prothymosin alpha, *PTMA* (P06454) 10/14 (71%) Capping protein, Arp2/3 and myosin-I linker protein 2, *CARMIL2* (Q6F5E8) ***Microbial proteins*** 11/23 (48%) DNA ligase B, *ligB, Klebsiella (K.) pneumoniae* (B5XTF0) 9/13 (69%) 60S ribosomal protein L37-A, *RPL37A, Saccharomyces* (*S.*) *cerevisiae* (P49166) 7/11 (64%) UPF0102 protein YraN, *yraN, Salmonella enterica* (A0A426WQ81)
UH-axSpA-IgA.3	28S ribosomal N3 (NR_146154.1)	Ribosomal RNA	(A)GKANDQRSWGRNDLNL FSNFKWVRSPARWRGAGR GMRVPSGPLLVSRTGAAG*	53	***Human proteins*** 17/32 (53%) Coilin, *COIL* (P38432) 17/27 (62%) Scaffold attachment factor B2, *SAFB2* (Q14151) 9/12 (75%) Enoyl-(acyl-carrier-protein) reductase mitochondrial, *MECR* (Q9BV79) ***Microbial proteins*** 14/28 (50%) Hydrogenase-1 large chain, *hyaB, E. coli* (P0ACD8) 20/47 (43%) Multidrug resistance protein MdtA, *mdtA, E. coli* (B1LNW7) 9/14 (64%) Uncharacterized protein YciO, *yciO, E. coli* (P0AFR4)
UH-axSpA-IgA.6	Zinc finger CCCH-type containing 3 (NM_015117.2)	mRNA, coding, no	(R)PAVADSGDGGKGDITAA DPPTAGSD*	26	***Human proteins*** 14/29 (48%) Ryanodine receptor 1, *RYR1* (P21817) 16/31 (51%) Uncharacterized protein C1orf167, *C1orf167* (Q5SNV9) 12/24 (50%) 3-oxoacyl-acyl-carrier-protein synthase, *OXSM* (Q9NWU1) ***Microbial proteins*** 9/15 (60%) L-lactate dehydrogenase, *lldD, Yersinia* (*Y.*) *pestis*, (A4TKI4) 11/25 (44%) Phthiocerol/phenolphthiocerol synthesis polyketide synthase type I PpsB, *ppsB, Mycobacterium tuberculosis* (Q7TXL9) 12/21 (57%) 30S ribosomal protein S3, *rpsC, Mycobacterium sp.* (A1UBP2)
UH-axSpA-IgA.7	kinesin family member 2A (NM_004520.5)	mRNA, 3’UTR	(T)RERDSDYE*	9	***Human proteins*** 7/8 (87%) SH2B adapter protein 3, *SH2B3* (Q9UQQ2) 6/6 (100%) Zinc finger protein 516, *ZNF516* (Q92618) 6/7 (85%) Probable global transcription activator SNF2L2, *SMARCA2* (P51531) ***Microbial proteins*** 5/6 (83%) ATP-dependent RNA helicase MSS116, *MSS116, S. cerevisiae* (P15424) 6/8 (75%) E3 ubiquitin-protein ligase HEL2, *HEL2, S. cerevisiae* (Q05580) 5/8 (63%) PHO85 cyclin-5, *PCL5, S. cerevisiae* (P38794)
UH-axSpA-IgA.8	Ubiquitin-conjugating enzyme E2D 3 (UBE2D3) genome (AF213884S1)	Intron, nc	(V)KHSLHEIFNTKPANGLS*	18	***Human proteins*** 8/9 (88%) Multimerin-1, *MMRN1* (Q13201) 6/6 (100%) N-lysine methyltransferase SETD6, *SETD6* (Q8TBK2) 9/14 (64%) Phosphatidylinositol 3,4,5-trisphosphate 5-phosphatase 1, *INPP5D* (Q92835) ***Microbial proteins*** 10/14 (71%) Ubiquitin carboxyl-terminal hydrolase 11, *RPN11, S. cerevisiae* (P43588) 8/13 (62%) Putative tyrosine-protein kinase in region, *NA, K. pneumoniae* (Q48452) 9/21 (43%) Carboxylic acid reductase, *car, Mycobacterium marinum* (B2HN69)
UH-axSpA-IgA.9	RNA 28S ribosomal 4 (NR_145822.1)	Ribosomal RNA	(G)KANDQRSWGRNDLNLFS NFKWVRSPARWRGAGRGM RVPSGPLLVSRTGAAG*	52	***Human proteins*** 17/32 (53%) Coilin, *COIL* (P38432) 17/27 (62%) Scaffold attachment factor B2, *SAFB2* (Q14151) 9/12 (75%) Enoyl-(acyl-carrier-protein) reductase mitochondrial, *MECR* (Q9BV79) ***Microbial proteins*** 14/28 (50%) Hydrogenase-1 large chain, *hyaB, E. coli* (P0ACD8) 20/47 (43%) Multidrug resistance protein MdtA, *mdtA, E. coli* (B1LNW7) 9/14 (64%) Uncharacterized protein YciO, *yciO, E. coli* (P0AFR4)
UH-axSpA-IgA.10	Histone deacetylase 3 (NM_003883.3)	mRNA, coding, Yes	(P)PEAPNEFYDGDHDNDK ESDVEI*	23	***Human proteins*** 22/22 (100%) Histone deacetylase 3, HDAC3 (O15379) 12/17 (70%) ADP-ribose glycohydrolase MACROD2, *MACROD2* (A1Z1Q3) 10/15 (66%) Neuroendocrine convertase 1, *PCSK1* (P29120) ***Microbial proteins*** 10/14 (71%) PHO85 cyclin-8, *PCL8, S. cerevisiae* (Q08966) 9/3 (69%) Bud site selection protein 14, *BUD14, S. cerevisiae* (P27637) 8/10 (80%) Uncharacterized protein YjgL, *yjgL, E. coli*

^a^Origin of the cDNA insert of the phage-displayed target.

^b^In-frame fusion of the cDNA coding region with the M13 gene VI: Yes/No. Translation of in-frame fusion results in expression of (part of) a human protein, whereas out-of-frame fusion results in a fusion construct with a non-physiological antigen sequence.

^c^Antigen sequence of the translated cDNA insert, with the first amino acid between parenthesis representing the transition between the M13 phagemid vector and the cDNA insert.

^d^Size of translated cDNA insert in amino acids.

Q Amber stop codon, which is translated into glutamine by the bacterial strain.

*Stop codon; mRNA, messenger RNA; nc, non-coding; UTR, untranslated region.

Interestingly, when comparing the antigen sequences to microbial sequences, we found that antigens UH-axSpA-IgA.1, UH-axSpA-IgA.3, UH-axSpA-IgA.6 to UH-axSpA-IgA.10 showed partial homology to proteins from several microbial species, including *Escherichia coli, Klebsiella pneumoniae, Saccharomyces cerevisiae*, and *Yersinia pestis* (see Table 2).

### Antibody reactivity against novel UH-axSpA-IgA antigens

The presence of IgA antibodies against the 7 UH-axSpA-IgA antigens was determined in individual plasma samples of early axSpA patients (*n* = 70, 55 axSpA patients from the plasma pools and 15 additional axSpA patients) from the UH cohort, and in matched HC (*n* = 88, HC cohort 1). Antibody reactivity against individual UH-axSpA-IgA antigens was present in 2.9% (2/70) to 8.6% (6/70) of early axSpA patients, and in 3.4% (3/88) to 6.8% (6/88) of HC (Table 3). The highest antibody reactivity in the samples of the early axSpA patients was seen for UH-axSpA-IgA.3 (8.6%) and UH-axSpA-IgA.10 (8.6%). The lowest reactivity in the HC samples was seen for UH-axSpA-IgA.1 (3.4%), UH-axSpA-IgA.3 (3.4%) and UH-axSpA-IgA.10 (3.4%).

**TABLE 3:** Antibody reactivity against individual UH-axSpA-IgA antigens, or a panel of antigens in early axSpA patients and HC from the UH cohort.

Antibody targets	axSpA UH n/N (%)	HC cohort 1 n/N (%)	LR+ (95% CI)	*P*-value
UH-axSpA-IgA.1	5/70 (7.1)	3/88 (3.4)	2.1 (0.5–8.5)	0.4677
UH-axSpA-IgA.3	6/70 (8.6)	4/88 (4.5)	1.9 (0.6–6.4)	0.3400
UH-axSpA-IgA.6	2/70 (2.9)	3/88 (3.4)	0.8 (0.1–4.9)	1.0000
UH-axSpA-IgA.7	2/70 (2.9)	6/88 (6.8)	0.4 (0.1–2.0)	0.3026
UH-axSpA-IgA.8	5/70 (7.1)	4/88 (4.5)	1.6 (0.4–5.6)	0.5109
UH-axSpA-IgA.9	4/70 (5.7)	4/88 (4.5)	1.3 (0.3–4.9)	0.7334
UH-axSpA-IgA.10	6/70 (8.6)	3/88 (3.4)	2.5 (0.7–9.7)	0.1860
At least one of 7	23/70 (32.9)	24/88 (27.3)	1.2 (0.8–1.9)	0.4862
UH-axSpA-IgA.1,3,10	17/70 (24.3)	9/88 (10.2)	2.4 (1.1–5.0)	0.0294

axSpA, axial spondyloarthritis; CI, confidence interval; LR+, positive likelihood ratio; n/N, number positive/number assessed; UH, University Hasselt.

Next, we investigated whether antibodies against a combination of specific UH-axSpA-IgA antigens could increase sensitivity while maintaining sufficient specificity in HCs. Therefore, the 3 antigens with the highest LR+ in early axSpA patients as compared to HC were combined into a panel: UH-axSpA-IgA.1, UH-axSpA-IgA.3, and UH-axSpA-IgA.10. Antibody reactivity against at least one of these 3 UH-axSpA-IgA antigens was found in 24.3% (17/70) and in 10.2% (9/88) of HCs (*p* = 0.0294), corresponding to a specificity of 89.8% and a LR+ of 2.4.

Moreover, clinical and disease characteristics (Supplementary Table 2) were compared between axSpA patients who tested positive for antibodies against this panel of 3 UH-axSpA-IgA antigens, and axSpA patients who were seronegative for this panel. We did not detect a significant difference in age, sex, HLA–B27 status, disease duration, treatment, erythrocyte sedimentation rate (ESR), CRP levels, or the presence of extra-articular manifestations. Furthermore, no significant difference was found for the disease activity scores Bath Ankylosing Spondylitis Disease Activity Index (BASDAI) (32) and Bath Ankylosing Spondylitis Functional Index (BASFI) (33) between early axSpA patients with, or without antibody reactivity against this panel of 3 UH-axSpA-IgA antigens.

### Antibody reactivity against a combined panel of UH-axSpA-IgA and UH-axSpA-IgG antigens was validated in the independent (Bio)SPAR cohort and, shows additional value for early axSpA diagnosis

Next, we determined whether IgA-isotype antibody reactivity in the UH-cohort against the 3 selected UH-axSpA-IgA antigens, UH-axSpA-IgA.1, UH-axSpA-IgA.3, and UH-axSpA-IgA.10 could be validated in a larger independent cohort of axSpA patients. To this end, we screened 164 samples from the (Bio)SPAR cohort. Furthermore, antibody reactivity was determined in additional control groups, including 109 age– and gender-matched HCs (HC cohort 2, including 17 HCs from HC cohort 1) and 66 patients with CLBP. The latter control group is of particular interest as clinical manifestations often overlap between axSpA patients and persons with CLBP in the early disease phase.

First, we found that the presence of antibodies against the 3 selected UH-axSpA-IgA antigens was confirmed in axSpA patients from the (Bio)SPAR cohort as antibody reactivity against individual antigens was observed in 4.3% (7/164) to 6.7% (11/164) of axSpA patients of the total (Bio)SPAR cohort (Table 4).

**TABLE 4:** Presence of antibodies against individual and combined UH-axSpA-IgA and UH-axSpA-IgG antigens in axSpA patients and controls.

Antibody targets	axSpA UH cohort			Bio(SPAR) cohort			CLBP	HC cohort 2
	n/N (sensitivity %)	*P*-value*	LR+* (95% CI)	n/N (sensitivity %)	*P*-value*	LR+* (95% CI)	n/N (specificity %)	n/N (specificity %)
UH-axSpA-IgA.1	5/70 (7.1)	0.2095	4.7 (0.6–39.3)	7/164 (4.3)	0.4446	2.8 (0.4–22.5)	1/66 (98.5)	6/109 (94.5)
UH-axSpA-IgA.3	7/70 (10.0)	0.7873	0.8 (0.3–2.2)	11/164 (6.7)	0.1912	0.6 (0.2–1.3)	8/66 (87.9)	5/109 (95.4)
UH-axSpA-IgA.10	5/70 (7.1)	0.0585	–	7/164 (4.3)	0.1966	–	0/66 (100.0)	6/109 (94.5)
UH-axSpA-IgG.4	5/70 (7.1)	0.0585	–	6/164 (3.7)	0.1861	–	0/66 (100.0)	1/109 (99.1)
UH-axSpA-IgG.8	4/70 (5.7)	0.3667	3.8 (0.4–32.9)	8/164 (4.9)	0.4523	3.2 (0.4–25.2)	1/66 (98.5)	4/109 (96.3)
UH-axSpA-IgA.1,10 UH-axSpA-IgG 4,8	18/70 (25.7)	0.0002	8.5 (2.1–35.2)	26/164 (15.9)	0.0065	5.2 (1.3–21.4)	2/66 (97.0)	15/109 (86.2)

axSpA, axial spondyloarthritis; (Bio)SPAR, Leuven Spondyloarthritis (Biologics); CI, confidence interval; CLBP, chronic low back pain; LR+, positive likelihood ratio; n/N: number positive/number assessed; UH, University Hasselt. *LR+ and *p*-values for diagnosis of axial SpA based on the presence of antibodies against UH-axSpA-IgA.1, UH-axSpA-IgA.3, UH-axSpA-IgA.10, UHaxSpA-IgG.4, and UH-axSpA-IgG.8 peptides, individually and combined, in axSpA patients compared to control subjects with CLBP.

Next, we investigated whether IgA-isotype antibody reactivity against UH-axSpA-IgA antigens could improve the diagnostic performance of IgG-isotype reactivity against previously identified UH-axSpA-IgG antigens (7). For this analysis, we determined antibody reactivity against 2 of these 3 UH-axSpA-IgG antigens, UH-axSpA-IgG.4 and UH-axSpA-IgG.8, as antibodies against them showed the highest sensitivity in a previous study in the UH and the (Bio)SPAR cohorts (7). Antibody reactivity against UH-axSpA-IgG.4 and UH-axSpA-IgG.8 was found in 7.1 and 5.7% of axSpA patients from the UH cohort and in 3.7 and 4.9% of the (Bio)SPAR cohort, respectively. We further explored whether a combination of antibodies against particular antigens could be of added value in distinguishing axSpA patients from persons with CLBP. From the 5 UH-axSpA-IgA/IgG antigens, we selected the 4 antigens with the highest LR+ in axSpA patients, compared to persons with CLBP: UH-axSpA-IgA.1,10 and UH-axSpA-IgG.4,8 (Table 4). Presence of antibody reactivity against at least one of these 4 antigens was significantly higher in axSpA patients from the UH cohort (25.7%, 18/70) compared to persons with CLBP (3%, 2/66) (*p* = 0.0002), with a corresponding LR+ of 8.5. In addition, antibody reactivity against at least one of these 4 antigens was also higher compared to HC (13.8%, 15/109), albeit not significantly (*p* = 0.0502, LR+ of 1.9). Antibody reactivity against at least one of these 4 antigens was also significantly higher in axSpA patients from the independent Bio(SPAR) validation cohort (15.9%, 26/164) compared to persons with CLBP (3%, 2/66) (*p* = 0.0065), with a corresponding LR+ of 5.2. Furthermore, in RA patients, we found that antibodies against at least one of the 4 UH-axSpA-IgA/IgG peptides were only present in 10.0% (6/60) of RA patients compared to 25.7% of the axSpA patients from the UH-cohort (18/70) (*p* = 0.0244) and 15.9% of the axSpA patients from the (Bio)SPAR cohort (26/164) (*p* = 0.3881). Data are shown in Supplementary Table 3.

As there is still an unmet need for biomarkers that can predict axSpA early after disease onset, we further investigated whether antibodies against this panel of 4 UH-axSpA antigens (UH-axSpA-IgA.1,10 and UH-axSpA-IgG.4,8) could be of added value in distinguishing early axSpA patients from persons with CLBP. Therefore, we performed a subanalysis on patients who had a maximum time of 5 years from diagnosis to study entry, including 70 patients from the UH cohort and 79 early axSpA patients from the (Bio)SPAR cohort (Table 5). Within all early axSpA patients, antibody reactivity against at least one of these 4 antigens was significantly higher (21.1%, 30/142) compared to persons with CLBP (3%, 2/66) (*p* = 0.0004), with a corresponding LR+ of 7.0. Finally, clinical and disease characteristics were compared between early axSpA patients who tested positive for antibodies against this panel of 4 UH-axSpA antigens (UH-axSpA-IgA.1,10 and UH-axSpA-IgG.4,8) and early axSpA patients who were seronegative for this panel (Table 6). We did not find a significant difference in age, gender, disease duration, treatment, BASDAI score, BASFI, ESR, CRP levels between early axSpA patients with, and those without antibody reactivity against this panel of 4 UH-axSpA-IgA/IgG antigens. The percentage of HLA-B27 positive patients was significantly higher in the group of patients who were seronegative for the panel for 4 UH-axSpA-IgA/IgG antigens.

**TABLE 5:** Presence of antibodies against UH-axSpA-IgA and UH-axSpA-IgG antigens in early axSpA patients from the UH and (Bio)SPAR cohorts compared to persons with CLBP.

Antibody targets	Early axSpA patients (UH + (Bio)SPAR)			CLBP
	n/N (sensitivity, %)	*P*-value*	LR+* (95% CI)	n/N (specificity, %)
UH-axSpA-IgA.1	7/142 (4.9)	0.4402	3.3 (0.4–26.0)	1/66 (98.5)
UH-axSpA-IgA.10	7/142 (4.9)	0.1000	–	0/66 (100.0)
UH-axSpA-IgG.4	9/142 (6.3)	0.0600	–	0/66 (100.0)
UH-axSpA-IgG.8	8/142 (5.6)	0.2775	3.7 (0.5–29.1)	1/66 (98.5)
UH-axSpA-IgA.1,10 UH-axSpA-IgG.4,8	30/142 (21.1)	0.0004	7.0 (1.7–28.3)	2/66 (97.0)

axSpA, axial spondyloarthritis; CLBP, chronic low back pain; CI, confidence interval; LR+, positive likelihood ratio; n/N, number positive/number assessed; *LR+ and *p*-values for diagnosis of axial SpA based on the presence of antibodies against UH-axSpA-IgA.1, UH-axSpA-IgA.10, UHaxSpA-IgG.4, and UH-axSpA-IgG.8 peptides, individually and combined, in axSpA patients compared to control subjects with CLBP.

**TABLE 6:** Clinical characteristics of the early axSpA patients from the combined UH and (Bio)SPAR cohort positive and negative for antibodies against the panel of 4 UH-axSpA-IgA/IgG antigens.

Clinical characteristics	Antibody positive (*n* = 30)^†^	Antibody negative (*n* = 112)^‡^	*P*-value
Age, mean (SD)	41.2 (12.3)	39.1 (12.4)	0.482
Male, n (%)	14 (46.7)	69 (61.6)	0.151
HLA-B27 positive, n (%)	15 (53.6)	75 (75.0)	0.036
Disease duration^a^ (years), mean (SD)	2.1 (1.7)	2.3 (1.8)	0.585
No medication use^b^, n (%)	4 (13.3)	9 (8.0)	0.474
NSAID use, n (%)	21 (77.8)	73 (74.5)	0.806
cDMARD use, n (%)	9 (33.3)	31 (31.6)	1.000
bDMARD use, n (%)	9 (30.0)	37 (33.0)	0.829
BASDAI, mean (SD)	4.3 (2.1)	4.3 (2.2)	0.912
Active disease (BASDAI > 4), n (%)	12 (52.1)	41 (50.0)	1.000
BASFI, mean (SD)	4.0 (2.6)	3.4 (2.5)	0.290
ESR (mm/h), mean (SD)	21.3 (28.0)	14.6 (17.1)	0.206
CRP (mg/L), mean (SD)	11.0 (19.0)	9.4 (14.8)	0.627
Extra-articular manifestations^c^, n (%)	7 (23.3)	22 (19.6)	0.807
• Inflammatory bowel disease, n (%)	5 (16.7)	13 (11.6)	0.536
• Psoriasis, n (%)	0 (0.0)	1 (1)	1.000
• Uveitis, n (%)	2 (6.7)	10 (9.2)	1.000

^a^Disease duration, time between diagnosis, and blood sampling.

^b^No medication use at the time of blood sampling.

^c^Including uveitis, inflammatory bowel disease, and psoriasis.

^†^Values were available for all characteristics except for HLA-B27 status (*n* = 28); NSAID use (*n* = 27); cDMARD use (*n* = 27), BASDAI (*n* = 23); BASFI (*n* = 23), ESR (*n* = 26); CRP (*n* = 26); psoriasis (*n* = 26).

^‡^Values were available for all characteristics except for HLA-B27 state (*n* = 100); NSAID use (*n* = 98); cDMARD use (*n* = 98); BASDAI (*n* = 82); BASFI (*n* = 82), ESR (*n* = 103); CRP (*n* = 106), psoriasis (*n* = 105); uveitis (*n* = 109).

axSpA, axial spondyloarthritis; BASDAI, Bath Ankylosing Spondylitis Disease Activity Index; BASFI, Bath Ankylosing Spondylitis Functional Index; bDMARD, biological disease modifying anti-rheumatic drug; cDMARD, conventional disease modifying anti-rheumatic drug; CRP, C-reactive protein; ESR, erythrocyte sedimentation rate; HLA-B27, human leukocyte antigen B-27; NSAID, non-steroidal anti-inflammatory drug; SD, standard deviation.

## Discussion

In this study, we identified antibodies against 7 novel UH-axSpA-IgA antigens. The presence of antibodies against 3 of these 7 UH-axSpA-IgA antigens was confirmed in the UH cohort and independent (Bio)SPAR cohort. Testing for the presence of antibodies against a combination of 2 of these UH-axSpA-IgA antigens and 2 of the previously identified UH-axSpA-IgG antigens with the highest biomarker potential strongly increased the potential diagnostic value to distinguish axSpA patients from persons with CLBP.

Here, we used the SAS screening technique to identify IgA antibodies against novel antigenic targets in early axSpA patients. These antigens originate from our human axSpA cDNA phage display library, previously constructed from synovial hip tissue from 3 axSpA patients (7). This library therefore forms an *in vitro* representation of the human synovial antigens expressed in these tissues, but also contains phage clones expressing non-physiological peptides, resulting from out-of-frame translation of the cDNA coding region, or from the translation of normally untranslated regions. Six out of 7 identified antigens express such non-physiological peptides, and these probably comprise epitopes that mimic *in vivo* antigen structures (mimotopes). Each of the 6 UH-axSpA-IgA antigens showed partial homology at the amino acid level to human proteins, involved in various biological processes such as transcription regulation [Scaffold attachment factor B2 (SAFB2)], calcium transport [Ryanodine receptor 1 (RYR1)], intracellular signal transduction [SH2B adapter protein 3 (SH2B3)], cell adhesion and platelet degranulation [Multimerin-1 (MMRN1)] and scaffolding [Coilin (COIL)]. In addition, the antigens targeted by the novel IgA antibodies also showed partial homology to several microbial proteins originating from micro-organisms such as *K. pneumoniae, S. cerevisiae*, and *E. coli.* This is in line with previous studies, which reported on the detection of antibodies of the IgA isotype against lipopolysaccharides of *S. enteritidis* and *S. typhimurium* (34), bacterial extracts of *K. pneumoniae* (35, 36) and cell wall components of *S. cerevisiae* (12) in patients with AS.

At present, it remains unclear whether antibodies targeting these 6 UH-axSpA-IgA antigens are antibodies resulting from an immune response against microbial antigens, or possible autoantibodies against self-antigens. Furthermore, it needs to be determined whether these antibodies have an effect on the proteins they target, and the related disease processes. Currently, none of the described homologous proteins (Table 2) have been directly implicated in axSpA etiology, although some links can be made with processes underlying axSpA pathology. Prothymosin, which shows sequence homology with the UH-axSpA-IgA.1 antigen, has been reported to enhance proliferation of fibroblast-like synoviocytes in a collagen-induced rat model of arthritis and has several immunomodulatory functions (37). In addition, SH2B3 was reported to regulate cytokine production *via* the Janus Kinase (JAK)- signal transducer and activator of transcription (STAT) pathway (38), which is shown to have important immune-regulatory functions in rheumatoid arthritis (RA) (39) and axSpA (40). Interestingly, gene expression analyses of peripheral blood mononuclear cells (PBMC) from RA patients revealed downregulation of the methyltransferase SETD6 compared to controls. Furthermore, SETD6 was expressed to a lower extent in RA patients who respond to TNF inhibitors compared to non-responders, thereby suggesting a role for NF-κB signaling (41). Even though the results from the BLAST search might give a suggestion on the possible identity of the *in vivo* antigens targeted by these anti-UH-axSpA antibodies, the exact identities of the mimotopes are subject for further study. Nevertheless, antibodies targeting such mimotope antigens can still result in interesting biomarkers with clinical relevance for the disease studied.

On the other hand, UH-axSpA-IgA.10 correctly expresses the final 22 amino acids of the C-terminus of human HDAC3, which has a total size of 428 amino acids. HDAC3 is a member of the class I subfamily of histone deacetylases, a class of enzymes that remove acetyl groups from lysine residues of both histone and non-histone proteins (42). Increasing evidence suggests a crucial role for HDAC3 in rheumatic diseases, as HDAC3 is required for type I interferon production and activation of signal transducers and activators of transcription 1 (STAT1) in fibroblast-like synoviocytes obtained from RA patients (43), and for LPS-induced activation of macrophages (44). Furthermore, HDAC3 was found to affect cytokine production in PBMCs of RA patients (45). In AS patients, HDAC3 expression was increased in PBMCs and inhibition of HDAC3 was associated with downregulation of TNF-1α expression (46), thereby indicating that HDAC3 can be a potential therapeutic target in the underlying pathological process of AS. Interestingly HDAC3 also seems to be involved in the regulation of bone formation and bone resorption *via* STAT1 (47), a key dysregulated process in SpA patients. However, at the moment, it remains unclear whether the function of HDAC3 might be affected by antibodies targeting HDAC3.

The presence of antibodies against each of 7 UH-axSpA-IgA antigens was initially determined in axSpA patients and HCs from the UH-cohort. Antibody reactivity in early axSpA patients ranges from 2.9 to 8.6%, whereas antibody reactivity in HC ranges from 3.4 to 6.8%. In order to increase the sensitivity of these individual antibody reactivities in axSpA patients, while preserving the specificity, we combined the antibody reactivity against 3 antigens with the highest LR+, UH-axSpA-IgA.1, UH-axSpA-IgA.3, and UH-axSpA-IgA.10 into a panel. Antibody reactivity against one of these 3 UH-axSpA-IgA antigens could be detected in 24% of the early axSpA patients with a corresponding specificity of 90% in HCs. We were not able to detect a significant difference in clinical characteristics between axSpA patients positive for IgA antibodies targeting our panel of 3 UH-axSpA-IgA antigens, and patients lacking these IgA antibodies. Within our study, these IgA antibodies do not seem to allow the identification of a particular subtype of axSpA patients, such as those with IBD. Similarly, in the study of De Winter et al. no clinical correlation between IgA anti-CD74 antibodies and IBD could be detected (17), and no correlation between the presence of IgA anti-CD74 antibodies and microscopic gut inflammation could be established in the Belgian (Be)Giant cohort of early axSpA patients (18). Furthermore, the presence of IgA antibodies against several microbial pathogens has not been correlated with a particular axSpA clinical phenotype.

In previous research, we showed that IgG isotype antibodies to the UH-axSpA-IgG.1, UH-axSpA-IgG.4, and UH-axSpA-IgG.8 antigens were significantly more present in early axSpA patients (14%) compared to patients with CLBP (5%). In this study, we investigated whether IgA antibody reactivity against the novel UH-axSpA-IgA antigens could be of added value to this previously identified anti-UH-axSpA-IgG antibody reactivity, in order to distinguish axSpA patients from persons with CLBP. By combining antibody reactivity against the 4 antigens with the highest LR+, we found that antibodies against the novel antigens UH-axSpA-IgA.1, UH-axSpA-IgA.10, and the previously identified UH-axSpA-IgG.4 and UH-axSpA-IgG.8 antigens, were significantly more present in a subset of early axSpA patients (25.7%) of the UH cohort compared to reactivity in persons with CLBP (3.0%). Antibody reactivity against this panel of 4 UH-axSpA antigens was also confirmed in the patients from the independent (Bio)SPAR cohort (15.9%, 26/164).

At present, it is still challenging to distinguish between axSpA patients and persons with CLBP at an early disease stage, as both groups have low back pain. We found that antibodies against the panel of 4 antigens were significantly more present in a subset of early axSpA patients (21.1%, 30/142) from the UH cohort and the (Bio)SPAR cohort, who had a maximum diagnosis time of 5 years. Comparison of this antibody reactivity between early axSpA patients and persons with CLBP, resulted in a LR+ of 7.0. This is higher than the LR+ of the currently used laboratory marker CRP (LR+ of 2.5) and lower than the genetic marker HLA-B27 (LR+ of 9.0) (48). Although some caution should be taken when interpreting the observed LR+ of 7.0 in our study, as a rather small control population of persons with CLBP (*n* = 66) is compared to early axSpA patients (*n* = 142). Validation of the antibody reactivity in a larger number of persons with CLBP is necessary to further confirm this LR+.

Furthermore, antibody reactivity against the combined panel of 2 UH-axSpA-IgA and 2 UH-axSpA-IgG antigens had a corresponding specificity of 86.2% in age- and gender-matched HC, corresponding to a LR+ of 1.5.

In conclusion, in combination with the previously identified antigens UH-axSpA-IgG.4 and 8, the identification of 2 novel UH-axSpA-IgA antigens, UH-axSpA-IgA.1 and UH-axSpA-IgA.10 results in the identification of a larger subset of axSpA patients, thereby showing some promising diagnostic biomarker potential.

## Data availability statement

Data are available upon request. The authors commit to making the relevant anonymized reactivity and patient data available for a specified purpose approved by the institution and VS, the principal investigator of the study, and with a signed data access agreement.

## Ethics statement

This study was conducted in accordance with the Declaration of Helsinki and was approved by the Local Ethics Committees of Jessa Hospital, UH, and Ziekenhuis Oost-Limburg (approval no. B243201422699). Biologic samples from patients and healthy controls were previously collected in different studies, which were approved by the Ethics Committees of Jessa Hospital, UH, and University Hospitals Leuven (approval nos. B322201215165, B243201627373, B32220083429, and B32220084074). All patients and healthy controls provided written informed consent, and all human biologic materials used in this study were kindly provided by the University Biobank Limburg (UBiLim) (25) and the Biobank of University Hospitals Leuven.

## Author contributions

PV, DQ, and VS: study concept and design. PR, PV, DQ, PG, JV, AA, FV, KV, and VS: acquisition of data. PR, PV, DQ, EL, KV, and VS: analysis and interpretation of data. All authors contributed to the article and approved the submitted version.
