# Supplementary material for: Antibodies of the immunoglobulin a isotype to novel antigens in early axial spondyloarthritis
Source: Front Med (Lausanne). 2023 Feb 8;9:1072453. doi: 10.3389/fmed.2022.1072453 (PMC9945964; doi:10.3389/fmed.2022.1072453)
Supplement: Supplementary file 1 [file Data_Sheet_1.docx]

Supplementary Data

**Supplementary table 1 Reactivity of IgA antibodies against 89 UH-axSpA-IgA antigens in 6 plasma pools of early axSpA patients, and 3 plasma pools of HC**

|  |  | | | **AxSpA** | | | | | | **HC** | | |
| --- | --- | --- | --- | --- | --- | --- | --- | --- | --- | --- | --- | --- |
|  |  | | | **Pool 1** | **Pool 2** | **Pool 3** | **Pool 4** | **Pool 5** | **Pool 6** | **Pool 1** | **Pool 2** | **Pool 3** |
| 1 | UH-axSpA-IgA.3 | | |  |  |  |  |  |  |  |  |  |
| 2 | UH-axSpA-IgA.6 | | |  |  |  |  |  |  |  |  |  |
| 3 | UH-axSpA-IgA.1 | | |  |  |  |  |  |  |  |  |  |
| 4 | UH-axSpA-IgA.9 | | |  |  |  |  |  |  |  |  |  |
| 5 | UH-axSpA-IgA.7 | | |  |  |  |  |  |  |  |  |  |
| 6 | UH-axSpA-IgA.8 | | |  |  |  |  |  |  |  |  |  |
| 7 | UH-axSpA-IgA.10 | | |  |  |  |  |  |  |  |  |  |
| 8 |  | | |  |  |  |  |  |  |  |  |  |
| 9 |  | | |  |  |  |  |  |  |  |  |  |
| 10 |  | | |  |  |  |  |  |  |  |  |  |
| 11 |  | | |  |  |  |  |  |  |  |  |  |
| 12 |  | | |  |  |  |  |  |  |  |  |  |
| 13 |  | | |  |  |  |  |  |  |  |  |  |
| 14 |  | | |  |  |  |  |  |  |  |  |  |
| 15 |  | | |  |  |  |  |  |  |  |  |  |
| 16 |  | | |  |  |  |  |  |  |  |  |  |
| 17 |  | | |  |  |  |  |  |  |  |  |  |
| 18 |  | | |  |  |  |  |  |  |  |  |  |
| 19 |  | | |  |  |  |  |  |  |  |  |  |
| 20 |  | | |  |  |  |  |  |  |  |  |  |
| 21 |  | | |  |  |  |  |  |  |  |  |  |
| 22 |  | | |  |  |  |  |  |  |  |  |  |
| 23 |  | | |  |  |  |  |  |  |  |  |  |
| 24 |  | | |  |  |  |  |  |  |  |  |  |
| 25 |  | | |  |  |  |  |  |  |  |  |  |
| 26 |  | | |  |  |  |  |  |  |  |  |  |
| 27 |  | | |  |  |  |  |  |  |  |  |  |
| 28 |  | | |  |  |  |  |  |  |  |  |  |
| 29 |  | | |  |  |  |  |  |  |  |  |  |
| 30 |  | | |  |  |  |  |  |  |  |  |  |
| 31 |  | | |  |  |  |  |  |  |  |  |  |
| 32 |  | | |  |  |  |  |  |  |  |  |  |
| 33 |  | | |  |  |  |  |  |  |  |  |  |
| 34 |  | | |  |  |  |  |  |  |  |  |  |
| 35 |  | | |  |  |  |  |  |  |  |  |  |
| 36 |  | | |  |  |  |  |  |  |  |  |  |
| 37 |  | | |  |  |  |  |  |  |  |  |  |
| 38 |  | | |  |  |  |  |  |  |  |  |  |
| 39 |  | | |  |  |  |  |  |  |  |  |  |
| 40 |  | | |  |  |  |  |  |  |  |  |  |
| 41 |  | | |  |  |  |  |  |  |  |  |  |
| 42 |  | | |  |  |  |  |  |  |  |  |  |
| 43 |  | | |  |  |  |  |  |  |  |  |  |
| 44 |  | | |  |  |  |  |  |  |  |  |  |
| 45 |  | | |  |  |  |  |  |  |  |  |  |
| 46 |  | | |  |  |  |  |  |  |  |  |  |
| 47 |  | | |  |  |  |  |  |  |  |  |  |
| 48 |  | | |  |  |  |  |  |  |  |  |  |
| 49 |  | | |  |  |  |  |  |  |  |  |  |
| 50 |  | | |  |  |  |  |  |  |  |  |  |
| 51 |  | | |  |  |  |  |  |  |  |  |  |
| 52 |  | | |  |  |  |  |  |  |  |  |  |
| 53 |  | | |  |  |  |  |  |  |  |  |  |
| 54 |  | | |  |  |  |  |  |  |  |  |  |
| 55 |  | | |  |  |  |  |  |  |  |  |  |
| 56 |  | | |  |  |  |  |  |  |  |  |  |
| 57 |  | | |  |  |  |  |  |  |  |  |  |
| 58 |  | | |  |  |  |  |  |  |  |  |  |
| 59 |  | | |  |  |  |  |  |  |  |  |  |
| 60 |  | | |  |  |  |  |  |  |  |  |  |
| 61 |  | | |  |  |  |  |  |  |  |  |  |
| 62 |  | | |  |  |  |  |  |  |  |  |  |
| 63 |  | | |  |  |  |  |  |  |  |  |  |
| 64 |  | | |  |  |  |  |  |  |  |  |  |
| 65 |  | | |  |  |  |  |  |  |  |  |  |
| 66 |  | | |  |  |  |  |  |  |  |  |  |
| 67 |  | | |  |  |  |  |  |  |  |  |  |
| 68 |  | | |  |  |  |  |  |  |  |  |  |
| 69 |  | | |  |  |  |  |  |  |  |  |  |
| 70 |  | | |  |  |  |  |  |  |  |  |  |
| 71 |  | | |  |  |  |  |  |  |  |  |  |
| 72 |  | | |  |  |  |  |  |  |  |  |  |
| 73 |  | | |  |  |  |  |  |  |  |  |  |
| 74 |  | | |  |  |  |  |  |  |  |  |  |
| 75 |  | | |  |  |  |  |  |  |  |  |  |
| 76 |  | | |  |  |  |  |  |  |  |  |  |
| 77 |  | | |  |  |  |  |  |  |  |  |  |
| 78 |  | | |  |  |  |  |  |  |  |  |  |
| 79 |  | | |  |  |  |  |  |  |  |  |  |
| 80 |  | | |  |  |  |  |  |  |  |  |  |
| 81 |  | | |  |  |  |  |  |  |  |  |  |
| 82 |  | | |  |  |  |  |  |  |  |  |  |
| 83 |  | | |  |  |  |  |  |  |  |  |  |
| 84 |  | | |  |  |  |  |  |  |  |  |  |
| 85 |  | | |  |  |  |  |  |  |  |  |  |
| 86 |  | | |  |  |  |  |  |  |  |  |  |
| 87 |  | | |  |  |  |  |  |  |  |  |  |
| 88 |  | | |  |  |  |  |  |  |  |  |  |
| 89 |  | | |  |  |  |  |  |  |  |  |  |
|  | |  |  |  |  |  |  |  |  |  |  |  |
|  | | >0,1 |  |  |  |  |  |  |  |  |  |  |
|  | | >0,25 |  |  |  |  |  |  |  |  |  |  |
|  | | >0,5 |  |  |  |  |  |  |  |  |  |  |
|  | | >1,0 |  |  |  |  |  |  |  |  |  |  |
|  | | >2,0 |  |  |  |  |  |  |  |  |  |  |

Antibody reactivity against each of the 173 phage-displayed antigens was determined in 6 plasma pools, each consisting of 10 early axSpA patients, and 3 plasma pools, consisting of 10 HC each. Immunoreactivity of pooled samples against these individual phage clones was examined using phage ELISA. Antibody reactivity in pooled samples against each phage-displayed UH-axSpA-IgA antigen is expressed as the difference (delta) of the average optical density (OD) signal using the respective phage-displayed antigen, and the average OD signal using the phage without antigen (OD (specific phage) - OD (empty phage)). The level of antibody reactivity is depicted in a grey scale. Of the 173 phage-displayed antigens, 84 did not show reactivity in the axSpA plasma pools and were not included in the heatmap. The other 89 phage-displayed antigens were ranked based on highest reactivity in the number of axSpA plasma pools combined with minimal reactivity in the number of HC pools. Based on this ranking, the top 7 was selected, which showed reactivity in maximally one HC pool, and at least 3 more axSpA pools than HC pools, and were further validated in individual plasma samples.

**Supplementary table 2 Clinical characteristics of early axSpA patients antibody positive or**

**antibody negative against a panel of 3 UH-axSpA-IgA peptides**

| Clinical  Characteristics | Antibody  Positive  (n=17)† | Antibody  Negative  (n= 53)‡ | p-value |
| --- | --- | --- | --- |
| Age (mean, SD) | 43.5 (13.3) | 42.8 (11.8) | 0.835 |
| Male (n, %) | 11 (64.7) | 28 (52.8) | 0.418 |
| HLA-B27 positive (n, %) | 11 (64.7) | 27 (54.0) | 0.574 |
| Disease duration in years^a^ (mean, SD) | 2.6 (1.3) | 2.9 (1.4) | 0.347 |
| No medication use^b^ (n, %) | 4 (23.5) | 7 (13.2) | 0.443 |
| NSAID use (n, %) | 11 (64.7) | 39 (73.6) | 0.543 |
| cDMARD use (n, %) | 5 (29.4) | 22 (41.5) | 0.409 |
| bDMARD use (n, %) | 4 (23.5) | 12 (22.6) | 1.000 |
| BASDAI (mean, SD) | 4.7 (1.8) | 4.6 (2.2) | 0.876 |
| Active disease (BASDAI>4) (n, %) | 7 (53.9) | 21 (53.9) | 1.000 |
| BASFI (mean, SD) | 4.7 (2.3) | 3.9 (2.6) | 0.351 |
| ESR, mm/h (mean, SD) | 13.3 (13.3) | 11.2 (16.6) | 0.422 |
| CRP, mg/L (mean, SD) | 7.0 (10.4) | 5.6 (7.8) | 0.755 |
| Extra-articular manifestations^c^ (n, %) | 5 (29.4) | 9 (17.0) | 0.304 |
| - Uveitis (n, %) | 2 (11.8) | 6 (11.3) | 1.000 |
| - IBD (n, %) | 2 (11.8) | 2 (3.8) | 0.246 |
| - Psoriasis (n, %) | 0 (0.0) | 1 (1.9) | 1.000 |

^a^Disease duration, time between diagnosis and blood sampling

^b^No medication use at the time of blood sampling

^c^Including uveitis, inflammatory bowel disease and psoriasis

^†^ Values were available for all characteristics except for BASDAI (n=13), BASFI (n=12), ESR (n=15) and CRP (n=15).

^‡^ Values were available for all characteristics except for HLA-B27 (n=50), BASDAI (n=39), BASFI (n=39), ESR (n=45) and CRP (n=47).

axSpA, axial spondyloarthritis; BASDAI, Bath Ankylosing Spondylitis Disease Activity Index; BASFI, Bath Ankylosing Spondylitis Functional Index; bDMARD, biological Disease Modifying Anti-Rheumatic Drug; cDMARD, conventional Disease Modifying Anti-Rheumatic Drug; CRP, C-Reactive Protein; ESR, Erythrocyte Sedimentation Rate; HLA-B27, Human Leukocyte Antigen B-27; NSAID, Non-Steroidal Anti-Inflammatory Drug; SD, Standard Deviation.

**Supplementary table 3 Presence of antibodies against individual UH-axSpA-IgA and UH-axSpA-IgG antigens in axSpA patients and controls**

| **Antibody targets** | **AxSpA patients** | |  | **Controls** | | | |
| --- | --- | --- | --- | --- | --- | --- | --- |
|  | UH cohort  n/N (%) | (Bio)SPAR cohort  n/N (%) |  | CLBP  n/N (%) | HC cohort 2  n/N (%) | RA  n/N (%) | |
| UH-axSpA-IgA.1 | 5/70 (7.1) | 7/164 (4.3) |  | 1/66 (1.5) | 6/109 (5.5) | | 0/60 (0) |
| UH-axSpA-IgA.3 | 7/70 (10.0) | 11/164 (6.7) |  | 8/66 (12.1) | 5/109(4.6) | | 5/60 (8.3) |
| UH-axSpA-IgA.10 | 5/70 (7.1) | 7/164 (4.3) |  | 0/66 (0.0) | 6/109 (5.5) | | 3/60 (5.0) |
| UH-axSpA-IgG.4 | 5/70 (7.1) | 6/164 (3.7) |  | 0/66 (0.0) | 1/109 (0.9) | | 2/60 (3.3) |
| UH-axSpA-IgG.8 | 4/70 (5.7) | 8/164 (4.9) |  | 1/66 (1.5) | 4/109 (3.7) | | 1/60 (1.7) |
| UH-axSpA-IgA.1,10  UH-axSpA-IgG 4,8 | 18/70 (25.7) | 26/164 (15.9) |  | 2/66 (3.0) | 15/109 (13.8) | | 6/60 (10.0) |

axSpA, axial spondyloarthritis; (Bio)SPAR, Leuven Spondyloarthritis (Biologics); CLBP, chronic low back pain; n/N: number positive/number assessed; RA, rheumatoid arthritis; UH, University Hasselt.


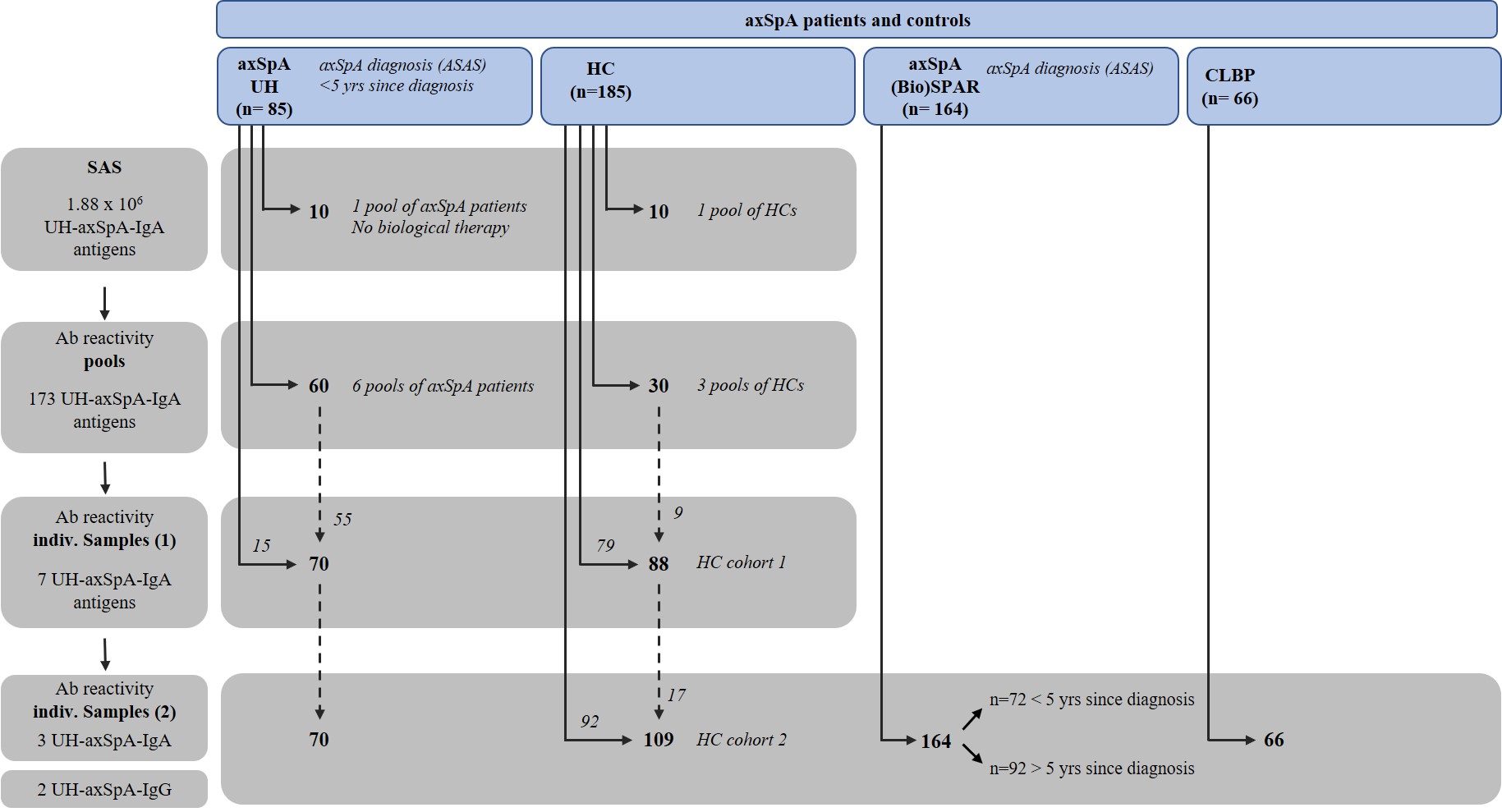
**Supplementary figure 1 Flow chart including the different steps of our screening and validation, and the corresponding patient and control populations**

axSpA, axial spondyloarthritis; (Bio)SPAR, Leuven Spondyloarthritis (Biologics); CLBP, chronic low back pain; HC, healthy control; n/N: number positive/number assessed; SAS, serological antigen selection; UH, University Hasselt.

Dotted lines indicate the samples that are shared between the different steps.

Full lines indicate the unique samples that are not shared between the different steps.

A human axSpA cDNA phage display library containing 1.88 x 10^6^ recombinant clones was used to identify novel UH-axSpA-IgA antigens in pooled plasma from 10 early axSpA patients and 10 healthy controls using serological antigen selection (SAS), which resulted in the identification of IgA antibodies against 173 novel antigens. Antibody reactivity against each of these 173 phage-displayed antigens was first determined in 6 additional plasma pools, each consisting of 10 early axSpA patients, and in 3 additional plasma pools, consisting of 10 HC each. Antibody reactivity against 7 selected UH-axSpA-IgA antigens was then determined in individual plasma samples from 70 early axSpA patients from the UH cohort (55 axSpA patients from the plasma pools and 15 additional axSpA patients), and 88 age- and gender-matched HC. Thereafter, antibody reactivity against 3 selected UH-axSpA-IgA antigens and 2 previously identified UH-axSpA-IgG antigens was determined in individual plasma samples from 70 early axSpA patients from the UH cohort, in 164 axSpA patients from the (Bio)SPAR cohort, 66 persons with CLBP, and an additional expanded set of 109 HC from the UH cohort, referred to as HC cohort 2.
